# Extent of knowledge and attitudes on plagiarism among undergraduate medical students in South India - a multicentre, cross-sectional study to determine the need for incorporating research ethics in medical undergraduate curriculum

**DOI:** 10.1186/s12909-022-03438-z

**Published:** 2022-05-18

**Authors:** Jeffrey Pradeep Raj, Shreeraam Venkatachalam, Rajkumar. S. Amaravati, Ramya Baburajan, Aswathy Maria Oommen, Jesin Elsa Jose, Rajad. R, Reshmi. R, Melvin George, Balaji Ramraj, Bhuvaneswari Gopalakrishnan, T. Suresh Kumar, Ahammed K. Saleem, Mohandas Rai, Vijay Subbaraju Penumutsa, Deepthi Rani Bodda, B. Lakshmi Prasanna, Guru Prasad Manderwad, Rajiv S, Basavaraj Bhandare, Prashanth Mada, Dilip Mathai, Rajalakshmi Aiyappan, Philip Mathew

**Affiliations:** 1 Department of Pharmacology, St. Johns Medical College, Bengaluru, 560034 Karnataka India; 2 Department of Orthopaedics, St. Johns Medical College, Bengaluru, 560034 Karnataka India; 3 Department of Anatomy, Government Medical College, Thiruvananthapuram, 695011 Kerala India; 4 Department of Anatomy, Government TD Medical College, Vandanam, Alappuzha, 688005 Kerala India; 5 Department of Physiology, Government TD Medical College, Vandanam, Alappuzha, 688005 Kerala India; 6 Department of Clinical Pharmacology, SRM Medical College Hospital & Research Centre, Kattankulathur, Chengelpet, 603203 Tamil Nadu India; 7 Indian Council of Medical Research, National Institute for Research in Tuberculosis, Chennai, 600031 Tamil Nadu India; 8 Department of Biochemistry, Government Vellore Medical College and Hospital, Adukkamparai, Vellore, 632011 Tamil Nadu India; 9 Department of Anatomy, Government Vellore Medical College and Hospital, Adukkamparai, Vellore, 632011 Tamilnadu India; 10 Department of Pharmacology, AJ Institute of Medical Sciences & Research Centre, Kuntikan, Mangaluru, 575004 Karnataka India; 11 Department of Pharmacology, Rangaraya Medical College, Kakinada, 533001 Andhra Pradesh India; 12 Department of Forensic Medicine, Kamineni Academy of Medical Sciences and Research Center, LB Nagar, Hyderabad, 500074 Telengana India; 13 Department of Microbiology, Kamineni Academy of Medical Sciences and Research Center, LB Nagar, Hyderabad, 500074 Telengana India; 14 Department of Pharmacology, Rajarajeswari Medical College and Hospital., No.202, Kambipura, Mysore Road, Bengaluru, 560 074 Karnataka India; 15 Department of Forensic Medicine & Toxicology, All India Institute of Medical Sciences, Hyderabad Metropolitan Region, Bibinagar, 508126 Telangana India; 16 Department of General Medicine, Apollo Institute of Medical Sciences & Research, Apollo Health City Campus, Jubilee Hills, Hyderabad, 500096 Telangana India; 17 Department of Community Medicine, Pushpagiri Institute of Medical Sciences & Research Centre, Thiruvalla, 689101 Kerala India

**Keywords:** Attitude, Plagiarism, Publication ethics, Research ethics

## Abstract

**Background:**

Undergraduate medical students in India participate in various research activities However, plagiarism is rampant, and we hypothesize that it is the lack of knowledge on how to avoid plagiarism. This study’s objective was to measure the extent of knowledge and attitudes towards plagiarism among undergraduate medical students in India.

**Methods:**

It was a multicentre, cross-sectional study conducted over a two-year period (January 2018 – December 2019). Undergraduate medical students were given a pre-tested semi-structured questionnaire which contained: (a) Demographic details; (b) A quiz developed by Indiana University, USA to assess knowledge; and (c) Attitudes towards Plagiarism (ATP) questionnaire.

**Results:**

Eleven medical colleges (*n* = 4 government medical colleges [GMCs] and *n* = 7 private medical colleges [PMCs]) participated. A total of *N* = 4183 students consented. The mean (SD) knowledge score was 4.54 (1.78) out of 10. The factors (adjusted odds ratio [aOR]; 95% Confidence interval [CI]; p value) that emerged as significant predictors of poor knowledge score were early years of medical education (0.110; 0.063, 0.156; < 0.001) and being enrolled in a GMC (0.348; 0.233, 0.463; < 0.001).The overall mean (SD) scores of the three attitude components namely permissive, critical and submissive norms were 37.56 (5.25), 20.35 (4.20) and 31.20 (4.28) respectively, corresponding to the moderate category.

**Conclusion:**

The overall knowledge score was poor. A vast majority of study participants fell in the moderate category of attitude score. These findings warrant the need for incorporating formal training in the medical education curriculum.

## Background

Research misconduct is defined by the office of Research Integrity USA as falsification, fabrication, or plagiarism while planning, conducting, or reviewing research, or while reporting the results of the research performed [1]. Among these, plagiarism is considered as the most serious breach of research integrity because it belittles someone’s original and honest contribution to the existing body of knowledge [2]. Plagiarism is nothing but copying another person’s words, ideas, or results, without giving appropriate credit [1]. Factors such as pressure to publish, easily available online texts and lack of awareness about the concept of plagiarism itself would lead a researcher to plagiarize. Pressure to publish papers has become a universal phenomenon [2] and Indian medical students are no different [3]. With the advent of internet, cyber plagiarism has become rampant not only in research but in various forms of non-research medical writing mostly due to lack of awareness about plagiarism [4].

Off late, the research aptitude among undergraduate (UG) medical students has been increasing with time [5]. In fact the apex research body of India, the Indian Council of Medical Research (ICMR) has been running the Short Term Studentship (STS) research program since 1979 and UG medical students undertake research studies for two months financially supported by the ICMR [6]. The number of STS recipients has been steadily increasing from 613 in 2007 to 1347 in the year 2019 [7]. The number of applications received for STS in the year 2019 (that corresponds to the study period) was approximately 10,000 [7] when the total number of UG medical seats available in India was approximately 80,000 [8]. Also, many UG medical research conferences are being organised in India as part of intercollegiate fest activities and students present posters or oral paper presentations [5]. These presentations and research publications are considered as important parameters by the program directors in developed nations such as the USA and the UK when UG students get matched to their residency programs [9]. As of 2017, around 69,000 Indian-trained physicians (equivalent to 6.6% of all registered medical practitioners in India) were working in countries such as the UK, USA, Canada, and Australia wherein these countries mandate compulsory enrolment in a residency program before they can practice [10]. Thus, a good number of UG medical students take up research projects these days. It is also not of place to mention that all UG students who get enrolled in a postgraduate training program in India have to submit a research project as dissertation before they become eligible for their exit exams [5].

Multiple studies in India and elsewhere have shown that plagiarism is widespread in education and academics and this has been documented under various settings like literature, art, engineering and science, including among medical and dental professionals [11–13] as well as students [14]. There is very little information on the extent of problem among UG medical students in India. A PubMed search using search string “Plagiaris* AND India*” retrieved 93 articles and there were no empirical research evaluating the knowledge and attitudes of plagiarism among UG medical students in India except for one study by Varghese et al*.*, in 2015 wherein the prevalence of those with poor knowledge (score ≤ 60%) was 63% (*n* = 265/423) and the knowledge score positively correlated with unfavourable attitudes [14]. Therefore, we proposed to do a larger multicentre study with a primary objective to estimate the prevalence of those with poor knowledge regarding plagiarism and explore the predictors of knowledge and attitudes towards plagiarism in UG medical students of India, thereby providing us an opportunity to recommend national level policy changes in UG medical education.

## Methods

### Ethics

The study protocol was approved by the Institutional Ethics Committee of the lead investigator (Vide reference number 214/2017 dated 12 September 2017) based on which administrative and ethics committee permissions were obtained from all the collaborating institutions. Further, a written informed consent was signed by all participants.

### Study design and eligibility criteria

It was a multicentre, cross-sectional study conducted over a two-year period from January 2018 – December 2019. Various Government medical colleges (GMC) and Private Medical Colleges / academic institutions (PMC) were approached based on convenience and necessary administrative permissions were sought. All undergraduate medical students of the institutions pursuing the Bachelor of Medicine, Bachelor of Surgery (MBBS) course including interns who agreed to participate in the study were counselled for participation in the study and those who did not consent were excluded. Those who were less than 18 years of age were also excluded as it would require a parental consent and most medical students live in hostels away from their families. The participants who did not answer all the questions in at least one of the sections (either knowledge or attitude), were excluded from the analysis as their filled questionnaire was considered invalid. Interns were included in the study population because internship in India is part of the UG degree program.

### Study setting

In India, there are two types of institutions based on their governance that offer medical courses. They are either run by the government (GMCs) or by private entities (PMCs). In any case, irrespective of whether a GMC or a PMC, the medical education curricula they follow is as prescribed by the erstwhile Medical Council of India (currently the National Medical Commission) [15]. The syllabus thus prescribed, does not make it mandatory for the UG medical students to get formally trained in research methodology and publication ethics. It is mostly done at the individual interest of the students more so as an “extra-curricular activity” [16].

### Sample size estimation

Assuming an estimated prevalence (p) of poor knowledge on plagiarism among medical undergraduate students as 63%, ^8^ the sample size calculated using the formula 4pq/ d^2^ with a relative precision (d) of 5% and q = 100-p, was 940. However, to account for the clustering effect of medical students belonging to the same medical college, the estimated sample size was multiplied using a design effect (DE). DE was calculated using the formula DE = 1 + ρ (cluster size—1) where cluster size (number of UG students in a medical college) was taken as 350 and ρ arbitrarily taken as 0.01. The DE thus calculated was 4.49 and the new sample size taking into effect the clustering was 4220.

### Study procedures

The students were met in their lecture halls as per their academic batches (groups of 20 – 30 students each) and the study was explained in detail after the printed informed consent form was distributed to the students. At the end of the briefing, students were given time to read the form and clarify if they had any doubts. Those who consented to participate in the study remained in the hall and they were handed over a semi-structured questionnaire comprising sections to capture their basic demographic details, enter answers for the 10 case vignettes assessing the extent of knowledge [17] and the validated attitudes towards plagiarism (ATP) questionnaire [18]. The extent of knowledge was assessed using a quiz developed for undergraduate students by Indiana University, USA (Freely available for use with due acknowledgement) [17]. The case vignettes comprised of an original version and a student’s version. Each participant had to compare both the versions and mark one of the three options namely: Word to word plagiarism, Paraphrasing plagiarism or no plagiarism. Subsequently the students marked their responses on a Likert scale of five for the various questions of ATP questionnaire [18]. The ATP questionnaire comprised of three scales to assess: (i) “positive” or permissive attitudes to plagiarism (items 1 to 12), (ii) “negative” or critical attitudes to plagiarism (items 13 to 19), and (iii) subjective or personal norms (items 20 to 29). Permissive attitude component assesses the extent to which one accepts the practice of plagiarism (Lower score is favourable) whereas critical attitude component assesses the extent to which one disapproves the practice of plagiarism (Higher score is favourable). The subjective norms component expresses one’s personal opinions regarding one’s own practices and those of others in their immediate circle thereby representing a possible ‘proxy for practice’ (Lower score is favourable). [18].

The students were guided section wise and were advised to wait for instructions on how to fill each section before they started filling to avoid making mistakes. The students were also reassured that the knowledge evaluation will be anonymous and their performance in this knowledge assessment will not affect any of their medical school grades, so that they do not feel the need to copy answers from another participant. The students were given enough time to complete the questionnaire and it was done during the official working hours of college so that the students do not fill the questionnaire in a hurried manner. Once the questionnaires were collected back from the participants, a short interactive session explaining the concept of plagiarism was conducted. A similar study was also conducted simultaneously among junior doctors with a different set of collaborating institutions [19].

### Data management

Data entry was done using Microsoft Excel (Publisher: Microsoft Corporation, Redmond, Washington, USA, 2016). Statistical analyses were performed using Statistical Package for Social Sciences (SPSS) Statistics for Windows, Version 20.0 (Publisher: IBM Corp., USA, 2011).

### Statistical analysis plan

The demographic characteristics were summarized using descriptive statistics. Data were tested for normal distribution using Kolmogorov Smirnov test. Poor knowledge score was defined was those scoring 60% and below [14]. The prevalence of those with poor knowledge score was expressed as proportions (95% Confidence intervals [CI]). A 2-way ANOVA was used to assess for differences in knowledge score across subsequent years of medical education and its interaction with the sector (whether GMC or PMC). The total number of participants in various categories of each component of the attitude was summarized using frequencies and percentages. Chi-squared test and post-hoc Beasley’s technique were used to analyse for differences in the number of participants across different categories of attitudes with regards to the type of medical college being enrolled in and their gender. A simple Bonferroni’s correction was applied for the p value for multiple comparisons pertaining to the type of medical college and hence, the new level of significance was *p* = 0.008. Univariate analysis was performed for the three attitude components and the hypothesized predictors of poor knowledge score using simple regression. Those predictors with a significance of *p* < 0.2 underwent multivariate analysis using linear regression model. The statistical significance for the study was set at *p* < 0.05 [19].

## Results

### Demography

A total of *n* = 15 medical colleges (*n* = 7 GMCs and *n* = 8 PMCs) across the 5 south Indian states and Union territory of Pondicherry were approached. A total of 11 medical colleges gave consent (*n* = 4 GMCs and *n* = 7 PMCs). The geographical distribution of the colleges include *n* = 2/11 from Karnataka, *n* = 3/11 (*n* = 1 GMC) from Tamil Nadu, *n* = 3/11 (*n* = 2 GMCs) from Kerala, *n* = 2/11 from Telangana and *n* = 1/11 (GMC) from Andhra Pradesh.

The number of students who were counselled were *n* = 4545 and the number of students who consented and filled the questionnaires were *n* = 4249 of which *n* = 66 were invalid. Thus, a total of *N* = 4183 participants were included in the final analysis. Besides this, students from one government medical college (*n* = 501) did not fill the knowledge questionnaire as administrative approval to evaluate the students for knowledge was not granted. Hence, they were not considered for the analysis of knowledge alone. The demographic characteristics of all *N* = 4183 participants are summarized in Table 1. Approximately 60% were female students and there was a good representation of students from all the years of medical school. A vast majority of them (approximately 97%) had either no publications in the past or were not currently involved in any research activities.

**Table 1:** Demographic characteristics of study participants

**Characteristic**		**Statistic**	**Government College** (*n* = 2131)	**Private College** (*n* = 2052)	**Total** (*N* = 4183)
Age		Mean (SD)	20.8 (1.8)	20.3 (2.2)	20.5 (2.0)
Gender	Male	n (%)	865 (40.6)	803 (39.1)	1668 (39.9)
	Female	n (%)	1266 (59.4)	1249 (60.9)	2515 (60.1)
Year of Study	One	n (%)	456 (21.4)	568 (27.7)	1024 (24.5)
	Two	n (%)	633 (29.7)	623 (30.4)	1256 (30.0)
	Three	n (%)	358 (16.8)	408 (19.9)	766 (18.3)
	Four	n (%)	552 (25.9)	321 (15.6)	873 (20.9)
	Internship	n (%)	132 (6.2)	132 (6.4)	264 (6.3)
Previous Publications	Yes	n (%)	44 (2.1)	34 (1.7)	78 (1.9)
	No	n (%)	2087 (97.9)	2018 (98.3)	4105 (98.1)
Currently involved in research	Yes	n (%)	60 (2.8)	96 (4.7)	156 (3.7)
	No	n (%)	2071 (97.2)	1956 (95.3)	4027 (96.3)

*SD* – Standard Deviation

**Table 2:** Predictors of poor knowledge score

Predictor	Univariate analysis		Multivariate analysis					
	β	*p* value	β	Std. β	95% CI	*p* value	VIF	EV (CIx)
Younger age	-0.017	0.234	Not included in analysis					
Male Gender	0.127	0.033	0.115	-0.032	-0.002, 0.231	0.054	1.001	0.390 (2.955)
Earlier years in education	0.107	< 0.001	0.110	0.076	0.063, 0.156	< 0.001	1.000	0.162 (4.586)
No Previous Publications	-0.146	0.473	Not included in analysis					
Not Currently involved in research	0.018	0.903	Not included in analysis					
Education in Govt Medical College	0.347	< 0.001	0.348	0.097	0.233, 0.463	< 0.001	1.002	0.045 (8.673)

*CI* = Confidence Interval; *VIF* = Variance inflation factor; *EV* = Eigen Value; *CIx* = Condition Index. *R* = 0.127; *R*^2^ = 0.016; adjusted *R*^2^ = 0.015

**Table 3:** Attitude towards plagiarism

Attitude	Category (Score range)	Frequency (%)
Positive / Permissive attitude (*n* = 4174)	Low* (12 – 28)	186 (4.5)
	Moderate (29—45)	3753 (89.9)
	High (46 – 60)	235 (5.6)
Negative / Critical Attitude (*n* = 4183)	Low (7 – 16)	705 (16.9)
	Moderate (17 – 26)	3208 (76.7)
	High* (27 – 35)	268 (6.4)
Subjective norms / Proxy for practice (*n* = 4178)	Low* (10 – 23)	165 (4.0)
	Moderate (24 – 37)	3771 (90.2)
	High (38 – 50)	242 (5.8)

^*^ Favourable attitude for academic integrity

### Knowledge score and its predictors

**Fig. 1:**
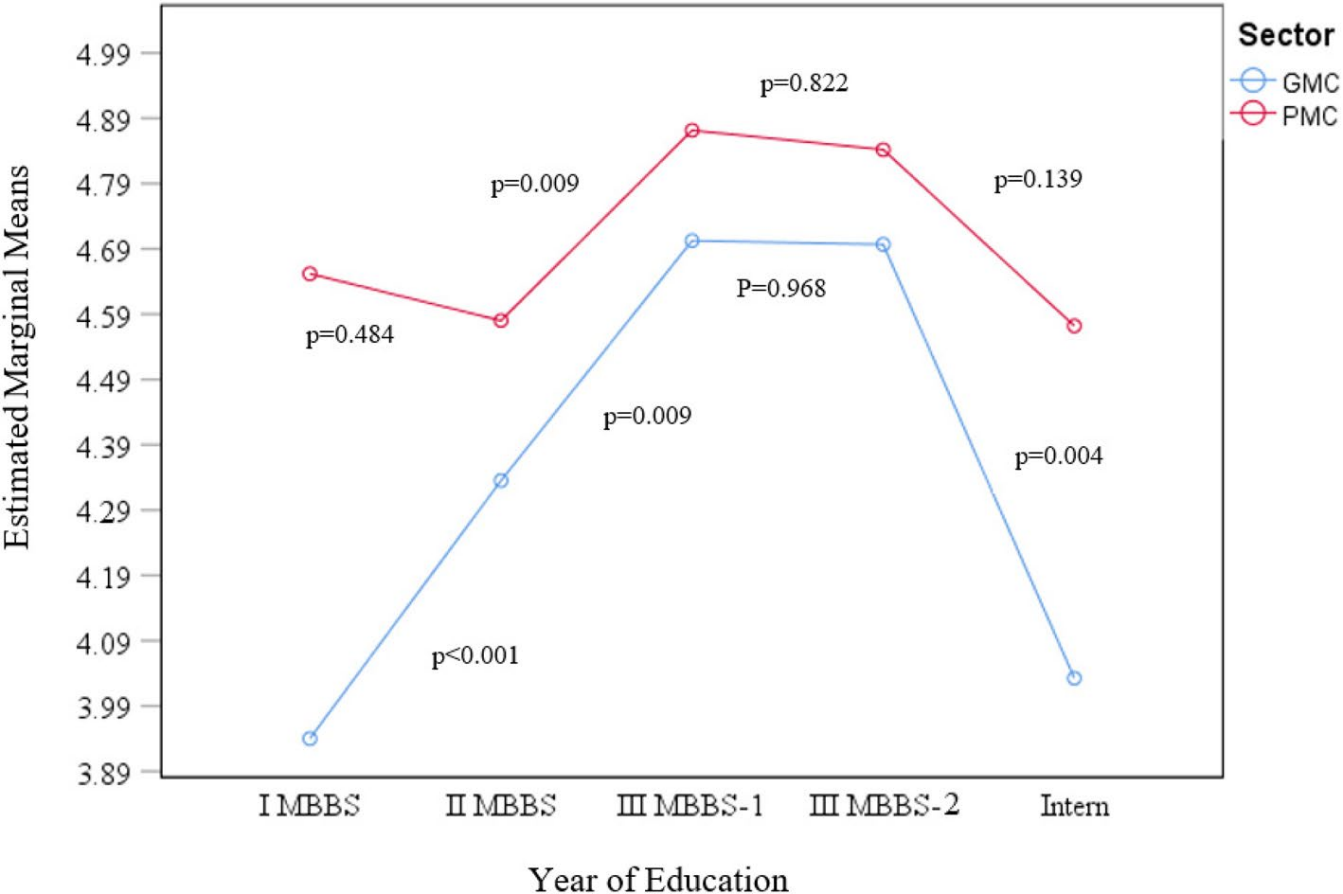
Trends in score over year of education in GMCs and PMCs (2-way ANOVA). The trends of scores (mean difference, *p* value) with every subsequent year in GMCs was as follows: from I MBBS to II MBBS, there was a significant increase in score (0.395, *p* < 0.001); from II MBBS to III MBBS part-1, there was another significant increase in score (0.367, *p* = 0.009); from III MBBS part-1 to III MBBS part-2 there was a non-significant decrease in score (-0.006, *p* = 0.968); from MBBS part 2 to internship, there was a significant fall in score (-0.664, *p* = 0.004). But in the PMCs, the scores of I MBBS were not different when compared to the other years. The trends of scores (mean difference, *p* value) with every subsequent year in PMCs was as follows: from I MBBS to II MBBS, there was a minor fall in score (-0.072, *p* = 0.484); from II MBBS to III MBBS part-1, there was a significant increase in score (0.291, *p* = 0.009); from III MBBS part-1 to III MBBS part-2 there was a non-significant decrease in score (-0.030, *p* = 0.822); from MBBS part 2 to internship, there was another non-significant fall in score (-0.270, *p* = 0.139)

The mean (SD) knowledge score was 4.54 (1.78) out of 10. The prevalence (Frequency [Proportion (95%CI)] of those with poor knowledge was *n* = 3181/3682 [86.4 (85.2, 87.5)]. The prevalence of those with poor knowledge score in GMCs was *n* = 1420/1630 [87.1 (85.4, 88.7) %] and that in PMCs was *n* = 1761/2052 [85.8 (84.2, 87.3) %]. The number (%) of participants who had scored nine or above, which is considered as the ideal score by Indiana university – the developers of the quiz, was a mere *n* = 18/3682 (0.05%). The mean (SD) score of participants from GMCs was 4.35 (1.87) and those from PMCs was 4.69 (1.70). The 2-way ANOVA that was conducted to examine the effect of year of study and the sector (whether GMC or PMC) of the medical college on the knowledge score suggested a significant interaction between the effects of year of study and sector of the medical college on the knowledge score, F(4, 3672) = 4.006, *p* = 0.003. The simple main effects analysis showed that the scores (mean difference, p value) of I MBBS (-0.712, *p* < 0.001), II MBBS (-0.245, *p* = 0.022) and Interns (-0.540, *p* = 0.039) in the GMCs score less when compared to those in PMCs but there was no difference for III MBBS part-1 and III MBBS part-2. In GMCs, the I MBBS, scored significantly less than all other years of education except for the interns whose score was almost similar to I MBBS. In PMCs, the I MBBS did not significantly differ from the scores of all other years of education including internship. The trends over change in scores over subsequent years of education in GMCs and PMCs individually is depicted in Fig. 1. The univariate and multivariate analysis of hypothesized risk factors for poor knowledge score are summarized in table -2. The factors (adjusted odds ratio [aOR]; 95% Confidence interval [CI]; *p* value) that emerged as significant predictors were early years of medical education (0.110; 0.063, 0.156; < 0.001) and being enrolled in a GMC (0.348; 0.233, 0.463; < 0.001).

### Attitudes towards plagiarism

Favourable attitudes are likely to represent better standards of academic integrity. This was noted in just a few student participants. The proportion (95% CI) of participants in the ideal categories for critical attitude, permissive attitude, and subjective norms respectively were 6.4% (5.7, 7.1), 4.4% (3.8, 5.1), and 5.8% (5.1, 6.5). Table-3 summarizes the category wise distribution of study participants in all the components of the ATP scale and it was observed that a vast majority fell in the moderate category in all the three components. The overall mean (SD) scores for critical attitude, permissive attitude, and subjective norms were 20.35 (4.20), 37.56 (5.25), and 31.20 (4.28) respectively. All the three means also corresponded to the moderate category. There was no significant difference with regards to gender distribution (*p* = 0.695, 0.106 and 0.233 respectively) across various categories for each attitude component. However, this difference was statistically significant with respect to being enrolled in GMC or PMC (*p* < 0.001 for all three analyses). Thus, when a post hoc analysis was performed, it revealed that 70.2% of those in the ‘high category’ for permissive attitudes were in the GMC (*p* < 0.0000001); 71% in the ‘low category’ for permissive attitudes were in the PMC (*p* < 0.0000001) and there was no difference between groups in the ‘moderate category’ (*p* = 0.676). On a similar note, 61.16% of those categorised as high for subjective norms belonged to the GMCs (*p* = 0.0011); 63.64% of those categorised as low belonged to the PMCs (*p* = 0.0001) and there was no difference between the groups with regards to moderate category (*p* = 0.96). However, with regards to critical attitudes, 56.7% of those categorised low (unfavourable attitude) were in the PMCs (*p* = 0.000007); 62.3% of those categorised as high were in the GMCs (*p* = 0.0001) with no difference between the groups in the moderate category (*p* = 0.080).

### Predictors of unfavourable attitudes

The univariate and multivariate analysis of the predictors of unfavourable attitudes for each of the three attitude components are described in Table 4. Of the hypothesized predictors (aOR; 95%CI; p value), education in GMC (-1.479; -1.135, -1.823, < 0.001) was the only significant predictor for positive attitudes score. Whereas, young age (0.073; 0.005, 0.140; 0.035), no previous publications (-1.794; -2.663, -0.925; < 0.001), not currently involved in research activities (-0.909; -1.594, -0.225; 0.009), education in GMC (-0.835; -1.112, -0.557; < 0.001) and poor knowledge score (-0.174; -0.250, -0.097; < 0.001) were the significant predictors for the score of critical attitudes. The significant predictors of the score for subjective norms were no previous publication (-1.194; -2.081, -0.306; 0.008), and Education in GMC (-0.665; -0.947, -0.382; < 0.001).

**Table 4:** Predictors of unfavourable attitudes towards plagiarism

**Predictor**	**Univariate analysis**		**Multivariate analysis**							
	β	*p* value	β	Std. β	95% CI	*p* value		VIF		EV (CIx)
Permissive attitudes*										
Younger age	-0.049	0.233	Not included in analysis							
Male Gender	0.132	0.426	Not included in analysis							
Earlier years in education	-0.079	0.228	Not included in analysis							
No Previous Publications	-0.731	0.181	-0.829	0.025	-1.903, 0.244		0.130		1.001	0.976 (1.640)
Not Currently involved in research	-0.489	0.256	Not included in analysis							
Education in Government Medical College	-1.416	< 0.001	-1.479	0.138	-1.823, -1.135	< 0.001		1.010		0.330 (2.819)
Poor knowledge score	-0.105	0.032	-0.066	0.022	-0.162, 0.029	0.174		1.010		0.068 (6.214)
*Critical Attitudes*^*#*^										
Younger age	0.098	0.003	0.073	0.035	0.005, 0.140	0.035		1.028		1.095 (2.028)
Male Gender	-0.286	0.031	-0.148	0.017	-0.426, -0.129	0.296		1.005		0.825 (2.336)
Earlier years in education	0.004	0.938	Not included in analysis							
No Previous Publications	-1.719	< 0.001	-1.794	-0.067	-2.663, -0.925	< 0.001		1.034		0.386 (3.415)
Not Currently involved in research	-1.087	0.001	-0.909	-0.043	-1.594, -0.225	0.009		1.028		0.126 (5.983)
Education in Government Medical College	-.757	< 0.001	-0.835	-0.097	-1.112, -0.557	< 0.001		1.026		0.062 (8.515)
Poor knowledge score	-0.197	< 0.001	-0.174	-0.073	-0.250, -0.097	< 0.001		1.011		0.005 (31.548)
*Subjective Norms*^*$*^										
Younger age	-0.037	0.260	Not included in analysis							
Male Gender	-0.057	0.674	Not included in analysis							
Earlier years in education	0.032	0.548	Not included in analysis							
No Previous Publications	-1.271	0.018	-1.194	.044	-2.081, -0.306	0.008		1.024		1.082 (1.579)
Not Currently involved in research	-0.679	0.052	-0.417	.019	-1.118, 0.283	0.243		1.023		0.822 (1.812)
Education in Government Medical College	-0.754	< 0.001	-0.665	.076	-0.947, -0.382	< 0.001		1.011		0.330 (2.861)
Poor knowledge score	-0.086	0.032	-0.070	.029	-0.148, 0.009	0.083		1.010		0.068 (6.301)

*CI* = Confidence Interval; *VIF* = Variance inflation factor; *EV* = Eigen Value; *CIx* = Condition Index. * *R* = 0.144; *R*^2^ = 0.021; adjusted *R*^2^ = 0.020, ^#^ *R* = 0.158; *R*^2^ = 0.025; adjusted *R*^2^ = 0.023. ^$^ *R* = 0.097; *R*^2^ = 0.009; adjusted *R*^2^ = 0.008

## Discussion

We report that the overall knowledge about plagiarism was low among undergraduate medical students. This observation is based on a sample that is representative of both PMCs and GMCs, all the years of medical school and includes a large proportion (60%) of female participants. The knowledge was better in students from PMCs [4.69 (1.70)] as compared to GMCs [4.35(1.87)] and the difference was statistically significant (*p* < 0.001). For all the three categories of attitude, the scores were moderate with 89.7% in the permissive group, 76.7% in the critical and 90.2% in the subjective attitude group.

The findings noted in this study were in line with the findings reported by Varghese et al [14]. We believe that it is because of this poor knowledge and unfavourable attitudes, the reported prevalence of those who had indulged in plagiarism of any kind in India is high – approximately 59% among medical students [20]. Further, it has been reported that this prevalence is equally high among nurses (43%) [20] as well as dental professional (40%) [21] and these findings could probably be considered as a continuum of the lack of awareness about plagiarism among the medical fraternity since medical field is the main branch that fuels healthcare academics.

The present study was done among undergraduate students, majority of whom (approximately 97%) were relatively unexposed to the concept of ‘research’ or a ‘publication’. This is because, many institutions in India, in general, do not give more emphasis to research [22]. Thus, lack of previous exposure to research may have resulted in less awareness on publication ethics. A study that evaluated causes of plagiarism found ‘*lack an understanding of what constitutes plagiarism*’ to be one of the reasons [23]. We observed that the scores in GMC were significantly low in the first year when compared to PMCs and increased drastically from the first year until the third year (III MBBS part 1) before dropping in the final and internship year. In contrast, the scores were relatively stable in the PMCs with a similar trend, although the differences were not statistically significant except for the significant increase between II MBBS and III MBBS-part 1. A possible reason could be the low emphasis on involvement in research activities, especially in GMCs and the students gradually pick-up with time [12] The later decline in the scores could be attributed to the inclination of most students in the concluding years towards preparation for competitive exams that test the technical knowledge rather than research aptitude. This suggests that there is a need to develop policies like mandatory trainings for students on publication ethics and research methodology at their 1^st^ year of MBBS course and a refresher course during their internship.

Surprisingly, studies indicate lack of knowledge of plagiarism in not only among students but also faculty members [24]. The institution policies and faculty have an important role to play in developing the ‘research culture’. The lack of focus on research and related practices at the institute level could be a reason why students remain largely unaware of these concepts until they start with post graduate education. The faculty here have an important role to play and in order to promote faculty development the Medical Council of India (now the National Medical Commission) has made in mandatory for faculty to publish research to be eligible for promotion [25]. However, this has resulted in a strategy driven by increasing the ‘quantity’ of papers published rather than focus on the ‘quality’. A study that analysed attitude of dental professionals towards plagiarism found that over 40% had indulged in the practice of plagiarism quite frequently [21]. This trend suggests that lack of culture to promote research integrity at the institute level could be another reason for the poor knowledge observed. This is complemented by cyber plagiarism which has now become the new normal, primarily because of its common occurrence in our day to day academic activities, making it difficult for the students to differentiate what is right and wrong [4].

The predictors of poor knowledge scores in this study were variables like early years of education and education in a GMC. It is estimated that with every additional year of medical education, the knowledge score would rise by an average 0.110 units. Similarly, in the event that a student moves from a GMC to a PMC, he/she is expected to have an average rise in score by 0.348 units. This is probably because, India’s public hospitals are usually overloaded with patients besides shortages in man-power and infrastructure wherein, patient well-being takes priority over research activities [26]. Besides faculty being busy with clinical work, a large majority of Indian institutions in general, lack a structured mentorship programme especially when it comes to research activities among undergraduate students [16].

Most of the students in the current study belonged to the moderate category for all the three components of attitudes with only a few (between 3 and 17%) in the most unfavourable attitude across all the three components. This is probably because, the students were relatively naïve to the concept of plagiarism and its consequence, as they did not have any formal training in research methodology and publication ethics which is the need of the hour. The study by Pallamparthy S et al.indeed states that at least 60% of the medical undergraduates who were surveyed proposed the inclusion of research methodology training in the syllabus [27]. A greater proportion of students with unfavourable permissive attitudes and subjective norms were seen in the GMCs while it was vice versa for critical attitudes. This finding was further endorsed by the multivariate regression analysis where, we found that students from GMC, in the event they migrate to a PMC their average attitude scores are expected to fall by 1.479, 0.835 and 0.665 units respectively in the positive, critical and subjective norm attitude component scores, all of which are favourable except for the critical attitudes score.

Further analysis of the predictors for unfavourable attitudes revealed that students who have a publication are likely to have their mean permissive and subjective norms attitude score, less by 0.829 and 1.194 units respectively, when compared to those without a publication. On a similar note, with every unit rise in the knowledge score, the permissive attitude score is expected to fall by an average 0.066 units, all of which are favourable. Our observation is in sync with some research papers that state ‘previous exposure’ as an important determinant of attitude towards research [28, 29]. For instance, Alhadlaq et al*.* from Saudi Arabia conducted a study among 551 medical students and observed that those who authored a scientific publication are more likely to have attitudes that disapprove plagiarism [30]. Previous experience about publication could contribute to the knowledge of students and studies in different divisions of science have shown that knowledge could be an important variable directing one’s attitude or behaviour [31].

However, with regards to the critical attitude score, we find that those who have a publication or who is currently involved in research, are likely to have their mean critical attitude score, less by 1.794 and 0.909 units respectively when compared to those who do not have a publication or not currently involved in any research activity. Accordingly, we find that for every unit rise in knowledge score, the critical attitude score falls by 0.174. All of these findings are not favourable and makes one think if there is a dampening effect on the attitude with research experience, due to the lack of strict institutional policies condemning plagiarism and awareness on the lack of sophisticated tools to detect complex forms of plagiarism at the institutional/university level. A study conducted by Javaeed et al*.* from the neighbouring nation, Pakistan, have indeed reported that a poor surveillance activity in detecting plagiarism at the institutional level, the absence of explicit policies to deal with issues pertaining to plagiarism, and a lack of awareness by the institution regarding the extent to which plagiarism prevails in their institute are important reasons that favours the act of plagiarism [32]. Further, a probable lack of awareness on the punitive consequences of being caught plagiarizing could be another reason as, such examples are not very common in a country like India.

This study has a few limitations. The total number of students who were included in the knowledge analysis was *n* = 3682, which fell short by 12.7% of the target sample size (*N* = 4220). However, given the large design effect incorporated while estimating the sample size and the narrow confidence intervals seen in the results of the present study, we could understand that the power of the study was not comprised for all computations based on knowledge assessment. Further, we have chosen participating institutions (clusters) by convenience which could have resulted in selection bias. However, a large sample size, a large cluster size and inclusion of all participants within the cluster is expected to have minimized this selection bias. Although, students from different institutions affiliated to different universities were enrolled, we were unable to objectively assess the influence of institute level policies on knowledge scores in the present study. Finally, the findings related to attitudes may not be generalizable because the overall knowledge on plagiarism was poor among the study participants and there were no other Indian studies to corroborate the findings from this current study. A study conducted among students with prior formal training on plagiarism and who are expected to be knowledgeable of this concept, would have shed light on the real predictors of poor attitudes toward plagiarism seen among the medical students of south India and would also confirm the findings from this study.

## Conclusion

Overall, the extent of knowledge towards plagiarism is low among the undergraduate medical students who participated in this study. Given the poor knowledge, the proportion of students with most favourable attitudes towards plagiarism was meagre and a vast majority belonged to the middle (moderate) category on all the three components of attitude namely permissive, critical and subjective norms. Since UG medical research is being given more importance these days for various reasons, and research has almost become inevitable in a medical graduate’s life, we recommend that formal training programs on research methodology and ethics be incorporated in the medical curriculum.

## Data Availability

The datasets generated and/or analysed during the current study are not publicly available due to lack of administrative sanctions from a few collaborating institutes, but are available from the corresponding author on reasonable request.

## Abbreviations

aOR: Adjusted Odds Ratio
ATP: Attitude Towards Plagiarism
CI: Confidence Intervals
DE: Design Effect
GMC: Government Medical College
ICMR: Indian Council of Medical Research
MBBS: Bachelor of Medicine, Bachelor of Surgery
PMC: Private Medical College
SD: Standard Deviation
SPSS: Statistical Package for Social Sciences
STS: Short term studentship
UG: Undergraduate

## Declarations

### Patient and public partnership

It was not appropriate to involve patients or the public in the design, or conduct, or reporting, or dissemination plans of our research.
